# High‐throughput, microscopy‐based screening and quantification of genetic elements

**DOI:** 10.1002/mlf2.12096

**Published:** 2023-12-18

**Authors:** Rongrong Zhang, Yajia Huang, Mei Li, Lei Wang, Bing Li, Aiguo Xia, Ye Li, Shuai Yang, Fan Jin

**Affiliations:** ^1^ CAS Key Laboratory of Quantitative Engineering Biology Shenzhen Institute of Synthetic Biology, Shenzhen Institutes of Advanced Technology, Chinese Academy of Sciences Shenzhen China; ^2^ Shenzhen Synthetic Biology Infrastructure Shenzhen Institute of Synthetic Biology, Shenzhen Institutes of Advanced Technology, Chinese Academy of Sciences Shenzhen China; ^3^ Chengdu Documentation and Information Center Chinese Academy of Sciences Chengdu China

**Keywords:** characterization methods, high‐throughput, robust genetic elements, synthetic biology

## Abstract

Synthetic biology relies on the screening and quantification of genetic components to assemble sophisticated gene circuits with specific functions. Microscopy is a powerful tool for characterizing complex cellular phenotypes with increasing spatial and temporal resolution to library screening of genetic elements. Microscopy‐based assays are powerful tools for characterizing cellular phenotypes with spatial and temporal resolution and can be applied to large‐scale samples for library screening of genetic elements. However, strategies for high‐throughput microscopy experiments remain limited. Here, we present a high‐throughput, microscopy‐based platform that can simultaneously complete the preparation of an 8 × 12‐well agarose pad plate, allowing for the screening of 96 independent strains or experimental conditions in a single experiment. Using this platform, we screened a library of natural intrinsic promoters from *Pseudomonas aeruginosa* and identified a small subset of robust promoters that drives stable levels of gene expression under varying growth conditions. Additionally, the platform allowed for single‐cell measurement of genetic elements over time, enabling the identification of complex and dynamic phenotypes to map genotype in high throughput. We expected that the platform could be employed to accelerate the identification and characterization of genetic elements in various biological systems, as well as to understand the relationship between cellular phenotypes and internal states, including genotypes and gene expression programs.

## Impact statement

The high‐throughput microscopy‐based platform, presented in this study, enables efficient screening of 96 independent strains or experimental conditions in a single experiment, facilitating the rapid identification of genetic elements with desirable features, thereby advancing synthetic biology. The robust promoters identified through this platform, which provide predictable and consistent control over gene expression under varying growth conditions, can be utilized as reliable tools to regulate gene expression in various biological applications, including synthetic biology, metabolic engineering, and gene therapy, where consistent system performance is required.

## INTRODUCTION

In recent decades, genetic screens for visual phenotypes have been highly successful genetic approaches^1^, ^2^, ^3^. However, among the various platforms for mapping genotype to phenotype, microscopy‐based methods are currently the only approach that allows for monitoring gene expression and/or cell behavior in single cells over time. This in‐depth analysis has proven to be a potent tool for understanding gene circuit dynamics, cell behavior heterogeneity, and broad‐spectrum phenotypes^3^, ^4^, ^5^, ^6^. It has become indispensable in biological engineering and synthetic biology, for applications such as library screening to develop new protein biosensors, synthetic enzymes, genetic elements, circuits, signaling pathways, and multicellular communities^2^, ^7^. We have seen the development of microscopy‐based, high‐content assays, which allow for parallel monitoring of cells possessing different genotypes. The utilization of fluorescently labeled proteins or fluorescent markers can visualize cellular and subcellular phenotypes. This method can image millions of cells from thousands of genetic variants and visually assess their phenotypes in a pooled format^8^, ^9^, ^10^, ^11^.

Recent advances in in situ sequencing methods allow for the visual reading of nucleic acid barcodes to map their genotypes^12^, ^13^, ^14^, ^15^, ^16^, ^17^, ^18^. When paired with fluorescence‐activated cell sorting^19^, ^20^, ^21^, ^22^, cells exhibiting desirable phenotypes can be isolated from pooled variants. However, these methods involve complex procedures, require expensive reagents, and need extensive computational pipelines. Using photoactivatable proteins to mark and sort cells necessitates additional genetic manipulation and high‐resolution light patterning^19^, ^20^.

Microscopy‐based approaches excel in analyzing the heterogeneous behavior of individual cells^6^, especially when screening for genetic elements with temporal or spatial properties, such as biosensor kinetics and gene expression dynamics^23^. Nonetheless, the preparation of samples for large‐scale experiments has been a significant bottleneck in classical microscopy investigations, limiting the method's throughput due to the labor‐intensive nature of sample preparation. Even with the optimization of commercial multi‐well dishes or cell microarrays for high throughput, the necessity of manual intervention to scan samples restricts the time resolution or the number of cells that can be analyzed^2^. Consequently, there is a pressing need for strategies capable of preparing hundreds of microscope samples simultaneously.

In our current study, we endeavored to augment the throughput of microscopy‐based experiments by devising a sampling device that facilitates large‐scale sample preparation. This tool is followed by a rapid, automated acquisition of images for all samples under the microscope. To demonstrate the versatility of our platform, we conducted a proof‐of‐concept study by screening and quantifying the activity of a library of natural promoters in *Pseudomonas aeruginosa*. Using the platform, we were able to simultaneously screen 96 promoter reporters in a high‐throughput manner. We identified robust promoters that showed stable functions across a range of growth and stress conditions in *P. aeruginosa* and *P. putida* KT2440. We further validated the robustness of the screened promoters by quantifying their temporal properties, such as growth rate, at the single‐cell level. The 96‐well agarose pad plate can be prepared using a standard protocol and is universally compatible with standard microscope object stages, making our platform easy to use for other groups. Because of the versatility and generality of our platform, we anticipate its wide utilization for accelerating the identification and characterization of genetic elements in a wide range of biological systems.

## RESULTS

### High‐throughput microscopy‐based platform for simultaneous analysis of hundreds of samples

**Figure 1:**
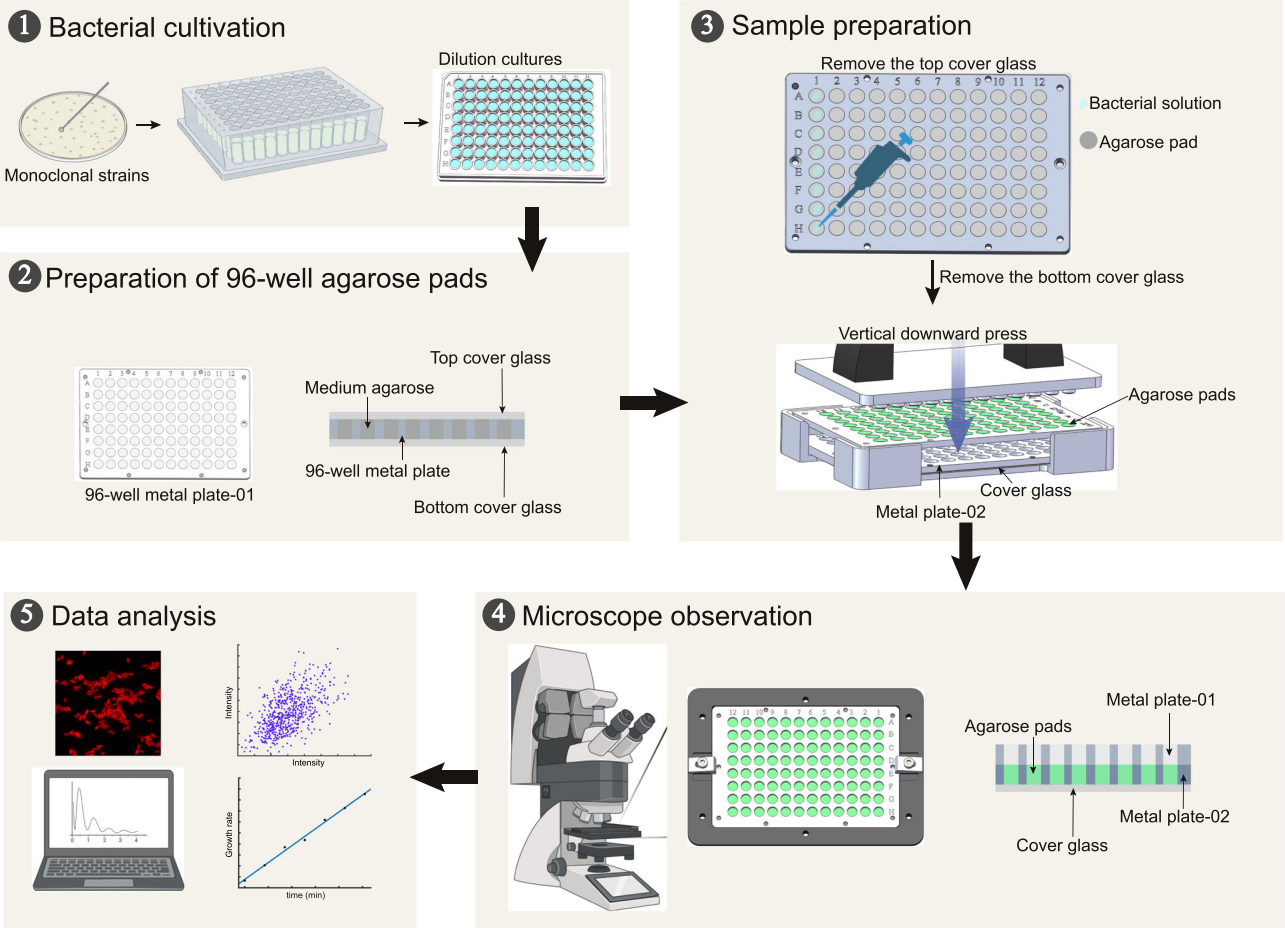
Sampling procedures for high‐throughput microscopy‐based platform for simultaneous analysis. (1) Bacterial cultivation: Bacterial strains recovered on LB (Luria‐Bertani medium) agar were scraped from the plates and resuspended into the 96‐deep‐well plate for cultivation and were further diluted into the 96‐well plate for following sample preparation. (2) Preparation of 96‐well agarose pads: The 96‐well agarose pad plates were prepared by sandwiching between the top and bottom cover glass in the first 96‐well metal plate. (3) Sample preparation: Tipping the diluted bacterial solution onto 96‐well agarose pad after removing the top slide glass, and sandwiching the bacteria between the agarose pad and cover glass with the downward press in vertically into the second metal plate after removing the bottom slide glass. (4) Microscope observation: The two metal plates harboring bacteria samples were anchored with magnetic force, then transferred onto the microscope for observation. (5) Data analysis: The acquired fluorescent images were processed and analyzed by using MATLAB with a self‐written code.

We designed a high‐throughput micro‐sampling device for bacteria that can simultaneously complete the preparation of an 8 × 12‐well agarose pad plate, thus facilitating the screening of 96 independent strains or experimental conditions in a single experiment (Figure 1, Step 1 and Step 3). The device employs a sandwich structure where bacterial cells are positioned between an agarose pad and a piece of cover glass. The thickness of the agarose pad is optimized to allow adequate air exchange while providing sufficient medium to foster bacteria growth (Figure S1, see Materials and Methods section for detailed sampling procedures). Moreover, the 96‐well agarose pad plate is specifically designed to be universally compatible with standard microscope object stages (Figure S2). When paired with a microscope equipped with a high‐speed camera and piezoelectric positioning stages, boasting response time of millisecond and microsecond ranges, our platform facilitates fast‐scanning imaging of all 96 samples automatically within a mere 15 min (Figure 1, Step 4). And with our streamlined experimental workflow, from sample preparation to one‐shot image scanning (Figure 1, Steps 2–4), the entire procedure can be completed within just 30 min, substantially boosting the screening throughput and efficiency. Finally, the resulting images are analyzed using custom‐designed software programs or codes (Figure 1, Step 5)^24^.

### Screening of a natural promoter library

**Figure 2:**
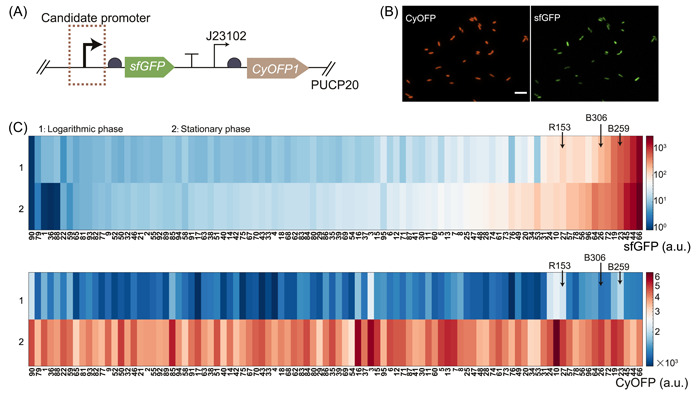
In situ high‐throughput characterization of promoter activity in natural promoter libraries using our 96‐well pad plate platform. (A) In the promoter library, the sfGFP fluorescent protein served as a quantifiable marker, while CyOFP1 fluorescent protein served as an internal control by fusing with a constitutive promoter J23102, in the vector PUCP20. (B) The representative fluorescent images in CyOFP1 and sfGFP channel. The scale bar is 5 μm. (C) Comparison of the sfGFP and CyOFP1 fluorescent intensity in the logarithmic phase and stationary phase for each promoter from the promoter library. According to the expression level of sfGFP, 96 promoters were initially screened from the initial library.

In a previous study, we constructed a dual‐color reporter library consisting of 3025 natural intrinsic promoters from *P. aeruginosa* ^25^. This library employed *sfGFP* as a quantifiable marker to measure the activity of each promoter, while the constitutive promoter J23102, fused to the gene of *CyOFP1* fluorescent protein, served as an internal control (Figure 2A). To evaluate the promoter activity with high throughput, we utilized our 96‐well pad plate platform to acquire dual fluorescent images of bacteria and analyze the resulting images to determine the fluorescent intensity (Figure 2B). Initially, we screened a subset of 96 promoters from the initial library (Table S1) and evaluated the fluorescence of sfGFP and CyOFP1 for each reporter in both logarithmic and stationary growth phases in FAB (an inorganic salt) medium (Figure 2C). For the constitutively expressed control gene, all strains exhibited a two‐fold higher fluorescence in CyOFP1 in the stationary phase than the logarithmic phase (Figure 2C), indicating that the growth conditions indeed influence protein expression, possibly due to changes in protein dilution time^26^. Regarding the sfGFP fluorescent reporter, we observed that a majority of promoters exhibited low activity, as indicated by sfGFP fluorescence levels below 50 a.u. (approximately 82%, 80 out of 96). To identify robust promoter candidates, we postulated that they should exhibit moderate to high activity while maintaining stable expression levels under varying growth conditions. As such, we screened 12 strains that displayed high sfGFP fluorescence greater than 50 a.u., with fluorescence differences no greater than 40% between the logarithmic and stationary phases (as indicated by the arrows in Figure 2C).

**Figure 3:**
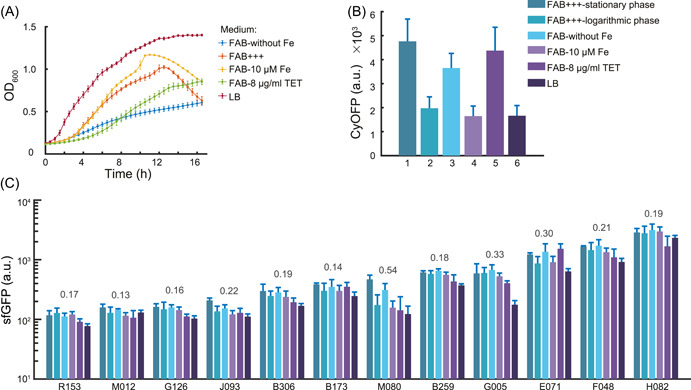
Characterization of robustness of 12 selected promoters from natural promoter library under different types of growth conditions. (A) The growth rate of bacteria varied greatly under five different culture conditions. OD_600_ of the selected promoters was measured in five types of growth conditions, including FAB without FeCl_3_ (FAB‐without Fe, marked with blue line), normal FAB media (FAB+++, marked with orange line), FAB with 10 μM iron (FAB‐10 μM Fe, marked with yellow line), FAB with antibiotic of tetracycline (FAB‐8 μg/ml tetracycline [TET], marked with green line), and rich medium LB (Luria‐Bertani medium, marked with brown line). (B) The average intensity of CyOFP1 of the selected promoters was measured in six different conditions, including FAB+++‐stationary phase, FAB+++‐logarithmic phase, FAB‐without Fe, FAB‐10 μM Fe, FAB‐8 μg/ml TET, and LB, respectively. (C) The average intensity of sfGFP of the selected 12 promoters (R153, M012, G126, J093, B306, B173, M080, B259, G005, E071, F048, and H082) was measured in the same conditions as (B). Data are presented as mean ± standard deviation (SD, *n*  =  3). The coefficient of variation (CV) values for each set of data are shown above the bars. The CV is calculated by the ratio of SD to mean. The greater the CV value, the greater the difference in promoter expression under different culture conditions, that is, the worse the promoter robustness.

We expanded our investigation to include five different types of growth conditions, which consisted of various culture media and stress conditions, such as rich medium, iron deprivation, and antibiotic addition (see Materials and Methods section for details). To assess the impact of endoplasmid on bacterial growth, we employed a microplate reader to continuously monitor the growth curves of 12 strains harboring different promoter reporter plasmids across eight distinct media conditions, as depicted in Figure S3. The observed overlap in the growth curves implied that the presence of endoplasmids, along with the different promoter reporter plasmids, did not significantly alter the growth dynamics of the bacteria under the tested conditions. We then measured the corresponding activity of the selected 12 promoters in *P. aeruginosa* under these conditions (Figure 3). In line with the results depicted in Figure 2C, the fluorescence intensity of CyOFP1 in all strains displayed considerable variability across different conditions (Figure 3B), with a high coefficient of variation (CV, the ratio of the standard deviation to the mean) of greater than 0.60.

In contrast, all 12 measured promoters showed moderate to high activity across all conditions, with about half of the promoters (7 of 12) exhibiting high conservation in expression levels across growth conditions in *P. aeruginosa*, as evidenced by a low CV of 0.2 for sfGFP fluorescence (Figure 3C). Together, by leveraging our 96‐well pad plate platform for screening, we successfully identified seven robust promoter candidates that exhibited minimal sensitivity to factors such as growth phase or culture medium composition in *P. aeruginosa*. The seven robust candidate promoters were screened as R153, M012, G126, B306, B173, B259, and H082. Their specific sequences are shown in Table S2.

### Characterization of promoter activity at the single‐cell level

**Figure 4:**
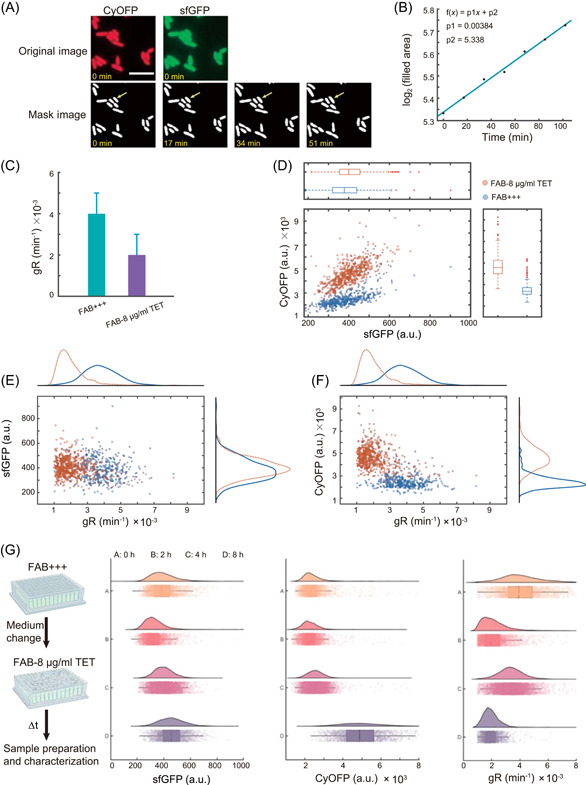
Efficient and quantitative characterization of the robustness of the selected promoter (B173) at single‐cell level using the high‐throughput microscopy‐based platform. (A) By identifying bacterial profiles in time‐lapse images, the strength of promoter expression can be calculated at the single‐cell level, thus characterizing the robustness of the selected promoter. The time‐lapse images were performed over a period of 2 h with 17‐min intervals with the mask images conducted from the original sfGFP and CyOFP1 images, where the yellow arrow indicates a tracked single cell. The scale bar is 5 μm. (B) Measuring the growth rate of single cell by fitting the time course data of cell area. (C) The mean cell growth rate was calculated by analyzing more than 10^3^ cells in FAB with antibiotic of tetracycline (FAB‐8 μg/ml TET), compared with that in normal FAB media. Data are presented as mean ± standard deviation (SD, *n* > 10^3^ cells). The relationships between the sfGFP and CyOFP1 intensity (D), between the growth rate and the sfGFP intensity (E), and between the growth rate and the CyOFP1 intensity (F) at the single‐cell level by generated scatter plots in FAB with antibiotic of tetracycline (FAB‐8 μg/ml TET) were visualized, compared with that in normal FAB media. (G) The single‐cell analysis of dual‐color fluorescence was performed at different culture times (0, 2, 4, and 8 h) with the histograms of the sfGFP intensity, CyOFP1 intensity, and growth rate, by changing the medium from normal FAB media to FAB with antibiotic of tetracycline. Δt represents the time cultured in medium containing TET.

One advantage of microscopy‐based screening is the ability to track temporal processes in single cells, and our platform is no exception. To illustrate this, we representatively performed single‐cell analysis of the B173 promoter reporter strain under normal and antibiotic‐added FAB medium conditions, as shown in Figure 4. In contrast to one‐shot image scanning, we performed time‐lapse imaging over a period of 2 h with 17‐min intervals (Figure 4A) and made single‐cell measurements of cell growth, such as growth rate, by fitting the time course data of cell area (Figure 4B). In all the experimental conditions we tested, *P. aeruginosa* grew the fastest in LB (Luria‐Bertani medium) (Figures S4 and 3A). We first conducted a quantitative characterization of the growth rate of *P. aeruginosa* in LB medium. As shown in Figure S5, we present accurate measurements of bacterial growth rate, indicating that the current image interval (17 min) employed in the *P. aeruginosa* strains meets the experimental requirements. We analyzed more than 10^3^ cells and found that the addition of antibiotics significantly decreased the mean cell growth rate (0.004 vs. 0.002 min^−1^, *p* < 0.001, Figure 4C), despite the final antibiotic concentration being below the minimal inhibitory concentration (MIC). Furthermore, we generated scatter plots to visualize the relationship between growth rate and fluorescence intensity at the single‐cell level (Figure 4D–F). The correlation coefficient between fluorescence intensity and growth rate was calculated at the same time. The fluorescence intensity of CyOFP1 expressed in strain B173 had a negative correlation coefficient of −0.52 with growth rate, while the correlation coefficient of sfGFP was close to zero. These results indicated that the expression of CyOFP1 decreased with the increase in growth rate (Figures 4F and S4), and the expression of sfGFP remained unchanged regardless of the growth rate (Figure 4E). In summary, the screened promoter B173 is robustness and is not affected by growth rate. To further validate the robust function of the promoter at different growth phases, we also performed single‐cell analysis of dual‐color fluorescence at different culture time. The results revealed a downward trend in the growth rate of bacteria as the culture time increased, indicating that the presence of tetracycline (TET) in the medium had a negative impact on bacterial growth. The histograms of the fluorescence intensity revealed that the fluorescence intensity of CyOFP driven by the constitutive promoter increased with culture time (0, 2, 4, and 8 h), whereas the fluorescence intensity of sfGFP driven by B173 promoter remained stable during cell growth (Figure 4G). Based on these single‐cell data, we confirmed that the activity of the B173 promoter is independent of the environment stress.

### Stable protein expression arises from consistent transcriptional activity

**Figure 5:**
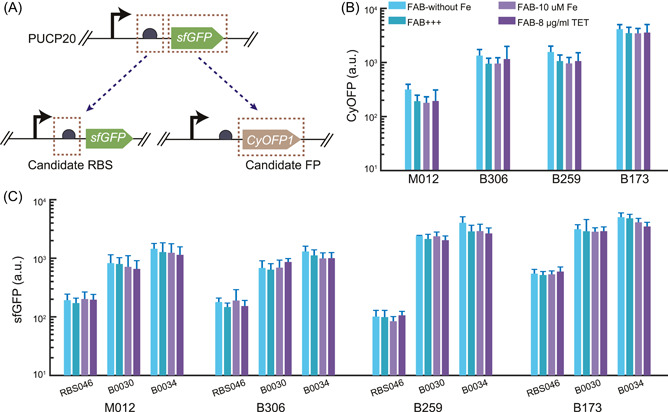
The stable protein expression driven by candidate robust promoters is independent of ribosome binding sites (RBS) or coding sequences. (A) Characterization of the four robust promoters from the promoter library with replaced RBS or reporter fluorescence protein. The schematic diagram of promoter with replaced RBS or reporter fluorescence protein (transfer to CyOFP1) are shown. (B) The CyOFP1 intensity of the four robust promoters (M012, B306, B259, and B173) with the replaced reporter fluorescence protein (CyOFP1) was measured in four different conditions, including FAB without FeCl_3_ (FAB‐without Fe), normal FAB media (FAB+++), FAB with 10 μM iron (FAB‐10 μM Fe), and FAB with antibiotic of tetracycline (FAB‐8 μg/ml TET). (C) The sfGFP intensity of the four robust promoters (M012, B306, B259, and B173) with the replaced RBS (RBS046, B0030, and B0034) was measured in four different conditions, including FAB‐without Fe, FAB+++, FAB‐10 μM Fe, and FAB‐8 μg/ml TET. Data are presented as mean ± standard deviation. The experiment was repeated three times.

Whereas protein expression involves the processes of transcription of a promoter and translation of information from a specific gene, we aimed to investigate whether stable expression of fluorescent proteins, driven by candidate robust promoters, depends on the ribosome binding site (RBS) or the coding sequence. We replaced the original RBS of four selected promoter reporters with three different constitutive prokaryotic RBSs of varying strengths and measured sfGFP expression in *P. aeruginosa* under the aforementioned five growth and stress conditions. We observed varying fluorescence intensity with different RBS strengths, but consistent fluorescence levels across the five different growth conditions for each RBS (CV < 0.17 for all reporters; Figure 5A,C). Furthermore, we replaced the original *sfGFP* coding sequence with that of another fluorescent protein, CyOFP1, and still observed stable fluorescence levels across different growth conditions (Figure 5A,B). These results suggest that the stable protein expression driven by the screening promoter arises from its robust transcriptional activity, rather than posttranscriptional processes.

### Identification of the core sequence responsible for stable transcriptional activity in candidate robust promoters

**Figure 6:**
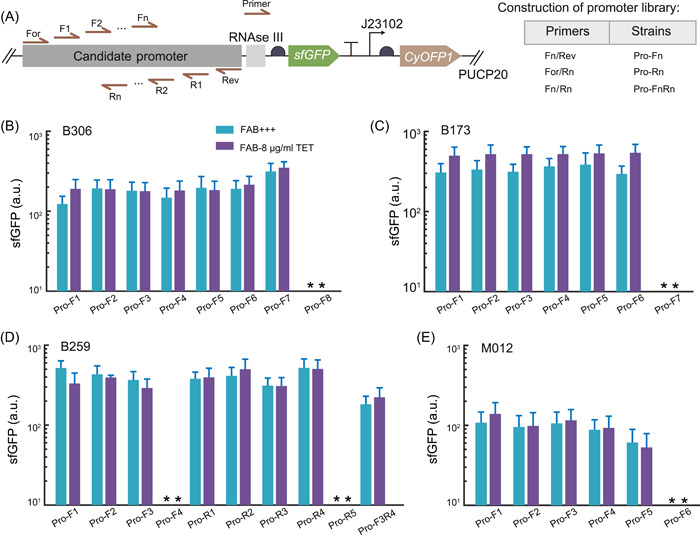
Identifying the core sequence responsible for stable transcriptional activity in candidate robust promoters. (A) The schematic diagram indicates the candidate promoters designed by truncating the promoters with different primers in vector PUCP20 and the construction of promoter library. (B–E) The sfGFP intensity of the candidate promoters (B306, B173, B259, and M012, respectively) was measured in the normal FAB media (FAB+++), and FAB with antibiotic of tetracycline (FAB‐8 μg/ml TET), where the truncated promoters referred to the promoter library in (A). The asterisk (*) indicates that the fluorescence intensity of bacteria is below the detection limit. Data are presented as mean ± standard deviation. Experiments were replicated three times.

When faced with unknown promoters and unclear regulatory mechanisms of promoter robustness, one approach is to construct a series of promoter truncated strains to systematically identify the core sequence of the promoters. By systematically truncating the promoter sequence, it becomes possible to identify essential regions or motifs that contribute to the promoter's activity. Due to the length of the initial promoter sequences being over 800 bp, redundancies may exist. We selected four screened promoters and progressively truncated them using different primer pairs (Figure 6A; see Materials and Methods section for details). This truncated promoter library was then used to identify the core sequence responsible for stable transcriptional activity. We screened the truncated promoter with the criterion of maintaining similar strength as the native promoter template while still exhibiting stable expression levels under varying conditions. Figure 6B–E shows the sfGFP expression levels of a partially truncated promoter library in *P. aeruginosa* under two conditions, and we ultimately identified the core sequences of the four selected promoters. These core sequences exhibited similar robustness but were much shorter, being less than 200 bp (Table S4).

### Measuring the performance of candidate robust promoters in alternative host

**Figure 7:**
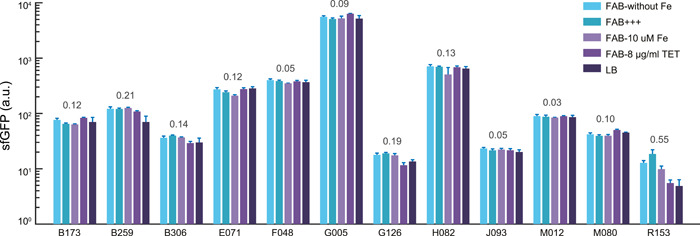
The screened promoters showed strong robustness in alternative host *Pseudomonas putida* KT2440. The activity of promoter in KT2440 strains under five different culture conditions was tested to characterize the robustness of promoter in KT2440. The sfGFP intensity of the candidate robust promoters (B173, B259, B306, E071, F048, G005, G126, H082, J093, M012, M080, and R153) was measured in different conditions in alternative host *P. putida* KT2440, including FAB without FeCl_3_ (FAB‐without Fe), normal FAB media (FAB+++), FAB with 10 μM iron (FAB‐10 μM Fe), FAB with antibiotic of tetracycline (FAB‐8 μg/ml TET), and rich medium LB (LB). The CV values for each set of data are shown above the bars. Data are presented as mean ± standard deviation. Experiments were replicated three times.

We proceeded to utilize our established 96‐well pad plate platform to conduct high‐throughput quantification of the fluorescence output generated from each of the candidate robust promoters in a different recipient, *P. putida* KT2440. We transformed the 12 promoter reporters from *P. aeruginosa* as depicted in Figure 2 and measured the sfGFP expression levels under five different growth and stress conditions (Figure 7). These promoters spanned nearly three orders of magnitude of fluorescence in *P. putida*, ranging from 40 to 4000 a.u. Moreover, most of all promoters exhibited stable functions across all conditions, with a low coefficient of variation (CV < 0.2) of fluorescence intensity among different conditions for each promoter (Figure 7). The results demonstrate the potential of these candidate promoters from *P. aeruginosa* to be expressed and applied in different host systems with flexibility.

## DISCUSSION

Current microscopy‐based experiments can achieve high throughput in imaging billions of cells and enable long investigation time spanning several hours^19^, ^20^. However, they often face a major limitation in sample preparation, namely the inability to measure hundreds of samples in a high‐throughput manner^2^, ^4^, ^5^, ^6^. In this study, we have addressed this limitation by developing a novel high‐throughput microscopy‐based platform. This platform makes use of a high‐throughput micro‐sampling device that enables the simultaneous preparation of an 8 × 12‐well agarose pad plate. This plate is then compatible with standard microscope object stages, allowing for the imaging screening of up to 96 independent strains or experimental conditions in a single experiment. By utilizing this platform, we were able to significantly increase the efficiency of our experiments, while also maintaining a high level of accuracy and precision in our measurements.

To highlight the flexibility, the platform was utilized to screen a library of natural promoters and identify robust promoter candidates that exhibited minimal sensitivity to factors such as growth phase or culture medium composition in *P. aeruginosa*. We then validate the robustness of these promoters in an alternative host, *P. putida* KT2440, and that all 12 candidate promoters retained stable functions across all tested conditions. These candidate promoters have the potential to be used in different host systems. Researching and applying robust promoters can enhance our understanding of gene regulation mechanisms and have important applications in synthetic biology, genetic engineering, and biomedical research.

Robust promoters represent a valuable tool for regulating gene expression in various biological applications, such as synthetic biology, metabolic engineering, and gene therapy. By providing more predictable and consistent gene expression levels under varying growth conditions, they can minimize the impact of environmental factors that could affect gene expression. This makes them particularly useful for designing and constructing synthetic gene circuits that require precise regulation of gene expression to achieve specific functions^27^. Moreover, robust promoters can enhance the production of useful biomolecules, such as therapeutic proteins or biofuels^28^, ^29^, by ensuring consistent and high‐level expression of the target genes. The use of a promoter library can also expand promoter diversity, improving DNA assembly efficiencies for larger and more complex gene circuits, while maintaining their evolutionary stability^30^, ^31^. In this study, we attempted to elucidate the factors contributing to the robustness of the screened promoters by employing a diverse range of strategies (Table S6). However, despite our diligent endeavors, a definitive mechanism explaining this robustness remains elusive. Our dedication to unraveling the underlying mechanisms will persist, and we aim to continue exploring and probing this intriguing aspect of promoter behavior to gain a more comprehensive understanding. Overall, robust promoters play a significant role in maintaining stable gene expression, reliable signal transduction, consistent gene expression, and mutation resistance. The identification and use of robust promoters have the potential to revolutionize the field of synthetic biology and enable the development of more efficient and reliable biological systems for a wide range of applications.

In light of the stable activity exhibited by these promoters, it is likely that they are regulated by different sigma factors that mediate nonequivalent levels of transcription in response to diverse milieu^27^, ^32^, ^33^. Alternatively, they may be subject to negative feedback coupling with growth rate or burden^34^, ^35^, ^36^, or other unknown mechanisms. Although unexplored in this study, future investigations could focus on unraveling the underlying mechanisms responsible for the stable gene expression of these candidate promoters.

Another key advantage of our established platform is that it allowed for the tracking of temporal processes in single cells, which enabled us to quantify cell features related to time series such as growth rate (Figure 4). This capability also allowed for analysis of gene expression dynamics, making high‐throughput screening of biosensors possible^37^. For instance, using our platform, we could screen fluorescent biosensors by monitoring the real‐time fluorescence changes in response to a specific messenger using microscopy in intact model organisms. With a capacity to screen 96 variants in high throughput, this approach could significantly increase the efficiency and speed of biosensor screening.

The platform utilized in this study has some limitations. While the concentration of agarose can be adjusted to allow for twitching motility when cells are grown under agarose pads, swimming motility cannot be assessed, which limits the screening of properties related to swimming. Additionally, although small‐molecule inducers or chemical perturbations can be easily added to the top of the pad while the bacteria are growing, their removal is difficult. However, the transparency of the agarose pads allows for the integration of optogenetic devices and genetic circuits to change or control input signals with light^38^. Another limitation of the platform is that while it allows for the tracking of temporal processes, tracking cell lineages for arbitrarily long timescales is not practical due to the limited nutrients and restricted space. As a solution, custom devices could be designed to continuously supply fresh medium to cells beyond the agarose, similar to the mother machine^39^.

Overall, the platform described here represents an advance in microscopy‐based screening approaches and has the potential to facilitate the identification of genetic elements with temporal or spatial properties such as biosensor kinetics and gene expression dynamics. We demonstrated the platform's flexibility by using it to screen and quantify robust promoters. The platform has the potential to be adapted for use with other microbial organisms, with some necessary adjustments, such as modifying the composition of agarose pads, to provide a powerful tool for high‐throughput genetic analysis. We anticipate that this platform's versatility and ability to enable quantification in high throughput will lead to its broad adoption in various fields, including biomedical sciences, bioengineering, and synthetic biology.

## MATERIALS AND METHODS

### Bacterial strains and growth conditions

The bacterial strains used in this study include *P. aeruginosa* strain PAO1 and *P. putida* KT2440. The screened promoters of initial library are based on the dual‐color reporter library of 3025 natural intrinsic promoters from *P. aeruginosa* from a previous study. The screened promoters and the corresponding gene name are listed in Table S1, and the detailed sequences of 12 analyzed promoters are enclosed in Tables S2. All bacterial culture experiments were conducted at 37°C. Strains carrying reporter plasmids were grown in the classical LB broth (10 g/l NaCl, 10 g/l tryptone, and 5 g/l yeast extract) supplemented with 30 μg/ml gentamicin overnight, following with a dilution into FAB medium with 30 mM glutamate as a carbon source under aerobic conditions, with the composition as follows: (NH_4_)_2_SO_4_, 2 g/l; Na_2_HPO_4_ ∙ 12H_2_O, 12.02 g/l; KH_2_PO_4_, 3 g/l; NaCl, 3 g/l; MgCl_2_, 93 g/ml; CaCl_2_ ∙ 2H_2_O, 14 g/ml; FeCl_3_, 1 μM; and trace metals solution (CaSO_4_ ∙ 2H_2_O, 200 mg/l; MnSO_4_ ∙ 7H_2_O, 200 mg/l; CuSO_4_ ∙ 5H_2_O, 20 mg/l; ZnSO_4_ ∙ 7H_2_O, 20 mg/l; CoSO_4_ ∙ 7H_2_O, 10 mg/l; NaMoO_4_ ∙ H_2_O, 10 mg/l; H_3_BO_3_, 5 mg/l) 1 ml/l. The FAB media with 10 μM FeCl_3_ or without Fe or with 8 μg/ml tetracycline were used when tested in different culture conditions. The shaker for cultivating bacteria was set at 800 rpm in a deep 96‐well plate.

### Construction of plasmids and strains

All plasmids and strains are listed in Table S3. All plasmids were constructed using basic molecular cloning techniques and Gibson assembly. All plasmids were sequenced and then transformed into PAO1 or KT2440 with standard protocols^40^, ^41^. The original plasmids used for calibrating the robustness of promoter contain two modules: *CyOFP1* driven by J23102 was used as an internal control, and *sfGFP* driven by fixed promoter was the reporter. To investigate whether stable expression of sfGFP, driven by candidate robust promoters (e.g., B173), depends on the RBS or the coding sequence, we replaced the RBS and the coding sequence sfGFP of candidate promoter in template plasmid of B173‐sfGFP‐J23012‐CyOFP1‐PUCP20 with RBS046/B0030/B0034 and CyOFP, respectively. B173‐CyOFP1‐PUCP20 was constructed by replacing *sfGFP* in B173‐sfGFP‐J23012‐CyOFP1‐PUCP20 with *CyOFP1* via Gibson assembly. The vector fragment B173‐PUCP20 without J23102‐CyOFP1 was obtained by PCR and then connected with the sfGFP fragment to construct the plasmid B173‐CyOFP1‐PUCP20. PCR fragments of sfGFP together with various RBS were ligated with linearized vector fragment B173‐J23012‐CyOFP1‐PUCP20 to generate plasmid B173‐RBS046/B0030/B0034‐ sfGFP‐J23012‐CyOFP1‐PUCP20.

To identify the core sequences responsible for stable transcriptional activity among candidate robust promoters, we constructed serial truncations of the promoter into dual‐color reporter vector and then validated the robustness of the truncated promoters in PAO1 cells. The schematic diagram of the construction of truncated promoter libraries is shown in Figure 6A. Taking the truncations of B173 promoter as an example, the detailed experimental process is as follows: A series of PCR primers were designed to amplify different truncations of the candidate robust promoters; the PCR fragments were cloned into reporter plasmid PUCP20 and placed upstream of sfGFP; the resultant plasmids were finally electroporated into PAO1 to generate the truncated promoter library.

### Preparation of 96‐well agarose pads

The 96‐well agarose pad plates were prepared by sandwiching between two (top and bottom) cover glass in the 96‐well metal plates. These plates have been electroplated on the surface to enhance their durability and resistance to corrosion, making them suitable for reuse. After each experiment, the device is subjected to two separate cleaning steps. First, the device is immersed in 75% alcohol and subjected to ultrasonic cleaning for 10 min. This helps to remove any residual substances or contaminants. Afterward, the device is cleaned with water using the same ultrasonic cleaning process. Once the cleaning process is complete, the device is dried and ready for reuse. To ensure accurate and high‐quality imaging results, fresh slides are necessary for each experiment. The cover glass was ultrasonic cleaned with 75% ethanol and Millipore water, respectively, for 10 min. The detailed process of preparing 96‐well agarose pads is shown in Figures S1 and S2. First, we glued a piece of cover glass on the bottom surface of sheet metal −01 and then flipped the metal plate so that the top surface was facing up. Second, the plastic choke plate was placed on the top surface of the metal plate that was locked in a waste tank. Third, bacterial culture medium containing 1% agarose was prepared, microwaved to melt completely, and then poured into the 96 holes in the metal plate. Next, we removed the choke plate and took out a clean cover glass. Then, let one side of the cover glass contact with the top surface of the agarose, and attach the cover glass tightly to the top surface of the metal plate to ensure that there are no air bubbles between the cover glass and metal plate. Finally, the device was left at room temperature until the agarose in the holes was completely solidified.

### Sampling procedures for high‐throughput microscopy‐based platform

Bacterial strains were recovered from −80°C fridge on LB agar (1.5% w/v) plates with 30 μg/ml gentamicin overnight at 37°C. Bacteria were scraped from the plates and resuspended in 1 ml of FAB media (96‐deep well plate) with 30 μg/ml gentamicin and 1 μM FeCl_3_. Strains were cultured overnight at 37°C and then diluted 100 times into 1 ml specific medium in 96‐deep well plates for 6‐h cultivation. To prevent the formation of bacterial clusters during the experiment, it is crucial to address the issue of high initial bacterial density in the liquid. Hence, it is essential to dilute the initial bacterial solution to a suitable concentration. The bacteria cultures from 96‐deep well plates were further diluted with fresh media before sampling onto the 96‐well agarose pad plate. The diluted bacterial solution was added to the samples using the 8‐Channel Pipettes, and the entire process took less than 5 min. If any holes are missed during the initial addition, additional drops of the bacterial solution can be added to those specific holes to ensure proper coverage. The diluted bacterial solution is tipped onto the 96‐well agarose pad after removing the top slide glass and the bacteria were sandwiched between the agarose pad and cover glass with the downward press in vertically by matching the two metal plates after removing the bottom slide glass. Then the 96‐well plates were transferred into the microscope for further observation and data analysis.

### Fluorescent image acquisition and data analysis

All qualitative images presented are representative of at least three independent duplicate experiments. Fluorescent images of sfGFP and CyOFP1 were acquired simultaneously by using two Zyla 4.2 Scmos cameras (Andor) with a fluorescence microscope (IX‐71; Olympus) equipped with a 100× oil objective. The fluorescence of both sfGFP and CyOFP1 was excited at 488 nm using a solid‐state light source (Lumencor Spectra X) and collected with emission filters of 520/528 and 583/522 nm, respectively. The sample arrangement and corresponding strain names are shown in Table S5. The microscope program was set to load 96 fields at once, each field being an independent strain or culture condition. Four pictures were collected in each field, and the number of bacteria in each picture was nearly 500. It took about 15 min to complete the image acquisition at one time point. To characterize the growth rate of bacteria, we collected images at least five time points with a time interval of 17 min.

Image processing was conducted by using MATLAB with a self‐written code. sfGFP and CyOFP1 images were aligned using a built‐in function (imwarp), and CyOFP1 images were used for bacterial cell identification. The single‐cell analysis of dual‐color fluorescence was conducted with specific MATLAB code, and scatter plots were generated for visualization with MATLAB. All experiment data are expressed as the mean ± SD.

## AUTHOR CONTRIBUTIONS

**Rongrong Zhang**: Conceptualization (lead); data curation (lead); funding acquisition (supporting); investigation (lead); writing—original draft (supporting). **Yajia Huang**: Data curation (lead); funding acquisition (supporting); investigation (supporting); project administration (lead); validation (lead); writing—original draft (supporting). **Mei Li**: Investigation (supporting); resources (supporting). **Lei Wang**: Investigation (supporting); resources (supporting). **Bing Li**: Investigation (supporting); resources (supporting). **Aiguo Xia**: Investigation (supporting); resources (supporting). **Ye Li**: Resources (supporting). **Shuai Yang**: Project administration (equal); software (equal); writing—original draft (equal). **Fan Jin**: Conceptualization (lead); funding acquisition (lead).

## ETHICS STATEMENT

This article does not contain any studies with human participants or animals performed by any of the authors.

## CONFLICT OF INTERESTS

The authors declare no conflict of interests.

## Supporting information

Supporting information.

## Data Availability

All data used during the study appear in this article.
